# Abnormally expressed miR-23b in Chinese Mongolian at high cardiovascular risk may contribute to monocyte/macrophage inflammatory reaction in atherosclerosis

**DOI:** 10.1042/BSR20180673

**Published:** 2018-11-16

**Authors:** Li-ping He, Xing-sheng Zhao, Le-ping He

**Affiliations:** Department of Cardiology, Inner Mongolia People’s Hospital, Huhhot 010020, China

**Keywords:** inflammatory response, miR-23b, Mongolian at high cardiovascular risk, macrophage, NF-κB

## Abstract

**Background**: The prevalence of coronary heart disease (CHD) appears to be high among Chinese Mongolians. MiR-23b has been proven to play a key role in atherosclerosis. The expression and role of miR-23b in the Mongolians at high cardiovascular risk were explored in the present study.

**Methods:** Forty cases of blood samples from the Mongolians at high cardiovascular risk were enrolled in the present study. The expression of miR-23b was quantified by quantitative real-time PCR. To induce monocytes differentiation into macrophages, HP-1 cells were cultured with phorbol 12-myristate 13-acetate. The level of inflammatory markers was determined by the enzyme-linked immunosorbent assay. The interaction between miR-23b and A20 was explored by the dual luciferase reporter assay.

**Results**: The expression of miR-23b in the Mongolian at high cardiovascular risk was higher than that in healthy Mongolian volunteers. Decrease in ATP-binding cassette transporter A1 caused by miR-23b is responsible for TC accumulation in the Mongolian at high cardiovascular risk. MiR-23b enhanced the oxidized low-density lipoprotein (oxLDL)-induced inflammatory response of THP-1 derived macrophage. MiR-23b regulated nuclear factor-κB (NF-κB) pathway through targeting A20. MiR-23b mediated oxLDL-induced inflammatory response of peripheral blood mononuclear cell in the Mongolian at high cardiovascular risk.

**Conclusion** MiR-23b enhanced oxLDL-induced inflammatory response of macrophages in the Mongolian at high cardiovascular risk through the A20/NF-κB signaling pathway, and thus contributing to atherosclerosis.

## Introduction

Coronary heart disease (CHD), also called ischemic heart disease, is featured with coronary arteries arteriosclerosis (CAA), which leads to stenosis and obstruction of lumen of the coronary, or coronarospasm, and final acute or temporary myocardial ischemia and anoxia [1]. CHD is commonly observed in middle-aged and aged people who are more than 40 years old, severely affecting the quality of life of patients and jeopardizing human health all over the world [2]. Generally speaking, hypertension, diabetes, hyperlipidemia, body mass index, smoking, and drinking are considered the main risk factors of CHD [3]. However, the pathogenesis of CHD is still too complex to elucidate.

Atherosclerosis refers to a process of chronic inflammation that characterized with plaque formation in the arterial wall, which is caused by cholesterol deposition, i.e. lipid accumulation and inflammatory response help to accelerate atherosclerosis [4]. Mononuclear phagocytes are essential cells involved in inflammatory response in CAA [5]. Macrophage inflammation is the principal element of atherosclerosis development, which has been found to aggravate atherosclerosis symptoms in animal model [6]. In addition, macrophage foam cell formation mediated by oxidized low-density lipoprotein (oxLDL) also contributes to the atherosclerotic plaque formation [7]. After oxLDL treatment in human THP-1 macrophages, the lipid accumulation and release of pro-inflammatory cytokines were elevated, which provided a model in research of arteriosclerosis progression mediated by macrophages [8]. As a central mediator of the inflammatory process, activation of the nuclear factor-κB (NF-κB) largely drives the inflammatory reaction of macrophage [9]. By suppressing NF-κB pathway, the macrophages inflammation in atherosclerosis mice could be effectively mitigated, showing a potential therapy for atherosclerosis [10]. The zinc-finger protein A20 is a key feedback inhibitor of the NF-κB pathway [11], playing a crucial role in immune responses of arteriosclerosis [12].

MicroRNAs (miRNAs) are a class of conserved non-coding RNAs that negatively regulate gene expression by targeting their mRNAs, widely participating in cell growth, differentiation, and apoptosis in various species [9,13]. Several miRNAs have been identified to be involved in atherosclerosis process [14]. When compared with the control group, a significantly increased expression of miR-23 in coronary artery disease (CAD) has been reported, suggesting the potential of miR-23 as a new biomarker in CAD diagnosis [15]. MiR-23b also has been claimed to participate in autoimmune inflammation in resident cells present in inflammatory lesions [16]. Even the effect of miR-23b on macrophage inflammation in atherosclerosis has not been revealed yet, the binding site between miR-23b and A20 was predicted by the bioinformatics method. Hence, it can be speculated that miR-23b may be implicated in NF-κB induced macrophage inflammation in atherosclerosis via interaction with A20.

Cardiovascular diseases based with stroke and CHD have been the leading cause of death in Chinese, and the Mongolian population is mainly located in northern areas of China. According to a previous report, the prevalence of CHD and the related death rates appeared to be high and have an increased tendency among Mongolians [17]. Hence, samples from the Mongolians at high cardiovascular risk were collected, and the expression of miR-23b in serum was investigated, to clarify the relationship between miR-23b and high cardiovascular risk in the Mongolians, and its role in macrophage inflammation mediated by NF-κB in atherosclerosis. The present study aimed to provide theoretical basis for atherosclerosis and CHD prevention through avoiding cardiovascular risk factors.

## Materials and methods

### Screening for high cardiovascular risk group in Chinese Mongolian

According to the guidelines for assessment and management of cardiovascular risk released in 2008 by the World Health Organization [18], the criteria of high cardiovascular risk are as follows. Subject who meets one of the following criteria should be judged with the high cardiovascular risk: (1) with the systolic blood pressure ≥ 160 mmHg, or with the diastolic blood pressure ≥ 100 mmHg; (2) with the low density lipoprotein-cholesterol ≥ 6 mM; (3) with the total cholesterol (TC) ≥ 8 mM; (4) with the rate between the TC and the high density lipoprotein-cholesterol (HDL-C) (TC/HDL-C) > 8; (5) with history of diseases, including myocardial infarction, percultaneous coronary intervention, and stroke.

### Clinical samples

A total of 150 cases of blood samples from the Mongolians at high cardiovascular risk were collected, among them 40 blood samples (high risk) were chosen randomly, and 20 cases of blood samples from healthy Mongolian volunteers were served as control. All the patients agreed and signed the documented informed consent for sample donation for study before sample collection. This research was approved by the ethics committee of the Inner Mongolia People’s Hospital and performed in accordance with the Helsinki Declaration.

### Quantitative real-time PCR

Quantitative real-time PCR (qRT-PCR) was performed to determine the expression of miR-23b and mRNA of MCP-1, TNF-α, and IL-1β. Total RNA was isolated from serum and monocytes by using the TRIzol reagent (Invitrogen) according to the manufacturer’s guidelines. Total cDNA was synthesized by using the iScript kit (Bio-Rad, PA, U.S.A.) for reverse transcription. By using Power SYBR Green RT-PCR Reagents (Applied Biosystems, U.S.A.), qRT-PCR analysis was performed on an ABI 7500 Fast Sequence Detection System (Applied Biosystems, U.S.A.). Data were collected and quantitatively analyzed with the 2^−ΔΔ*C*^_t_ method.

### Cell culture and differentiation induction

The human monocytic cell line THP-1 was purchased from the American Type Culture Collection (ATCC, U.S.A.). Cells were cultured at a density of 10^6^/ml in RPMI 1640 medium supplemented with 10% fetal bovine serum, 10 mM HEPES (Sigma), 1% pen/strep antibiotics, and incubated in a 5% CO_2_ incubator at 37°C. About 100 nM phorbol 12-myristate 13-acetate (PMA) (Calbiochem, U.S.A.) was added into THP-1 cells and maintained for 24 h to induce monocytes differentiation into macrophages.

### Cell transfection

To investigate the role of miR-23b expression in the macrophage differentiated from THP-1 cells, miR-23b mimic was obtained from GenePharma Co. Ltd (Shanghai, China) and was transfected into the above THP-1 derived macrophage. After 6 h, oxLDL (50 μg/ml) was introduced to stimulate the THP-1 derived macrophage, and the mRNA expression and protein level of inflammatory factors (TNF-α and IL-1β) and chemotactic factor (MCP-1) were determined 24 h later.

### Enzyme-linked immunosorbent assay (ELISA) of inflammatory markers

After transfected with miR-23b mimic for 6 h, PMA-differentiated THP-1 cells were incubated with oxLDL for another 24 h. The level of TNF-α, IL-1β, and MCP-1 in culture supernatants was determined by using Sandwich Enzyme Immunoassay kits (R&D Systems Europe Ltd, U.K.) according to the manufacturer’s instructions.

### Western blotting for protein level

After transfected with miR-23b mimic for 6 h, PMA-differentiated THP-1 cells were incubated with oxLDL for another 24 h. The protein from the cytoplasm and nuclear was separately extracted by using a Nuclear and Cytoplasmic Protein Extraction Kit (Sangon Biotech, Shanghai) as previously described [19,20]. Cytoplasmic protein was separated by lysis of pelleted cells in a neutral pH buffer containing 10 mM HEPES, 10 mM KCl, 0.1 mM EDTA, 0.1 mM EGTA, 0.5% NP-40, and a mixture of proteinase inhibitors including serine, cysteine, and metalloproteases (Complete, Roche-Boehringer-Mannheim, Germany). Pelleted cell nuclei were then lysed on ice in a hyperosmolar neutral pH solution with 20 mM HEPES, 0.4 M NaCl, 1 mM EDTA, 1 mM EGTA, and 10% proteinase inhibitors mixture. Protein extract concentration was determined spectrophotometrically at 540 nm in 96-well plate using BCA Protein Assay Reagents (Pierce, IL). Western blots were performed for the analyses of cytoplasm and nuclear protein level in THP-1 derived macrophages. In brief, the cytoplasmic and nuclear protein extracts were pre-treated for 10 min at 70°C in gel loading buffer containing detergent and reducing agent before separated by SDS-polyacrylamide gel electrophoresis. Then proteins were transferred onto PVDF membranes (Bio-Rad), and the membranes were incubated with the indicated primary antibodies in a blocking buffer and with a secondary HRP-conjugated antibody. The blots were developed using the enhanced chemiluminescence method (ECL from Amersham-Pharmacia, U.K.). Protein levels were normalized to β-actin and bands were quantified with a BioRad Personal Molecular Imager.

### Dual luciferase reporter assay

To investigate the regulatory mechanism of miR-23b on NF-κB, the interaction between miR-23b and A20 was explored by the dual luciferase reporter assay. The wild-type 3’-UTR (UTR WT) DNA segments of A20 containing the predicted miR-23b binding site were inserted into pmirGLO vector (Promega, U.S.A.), with the mutant 3’-UTR (UTR Mut) served as control. The recombinant vectors and miR-23b mimic (or its negative control, miRNC), or miR-23b inhibitor were co-transfected into HEK-293 cells by Lipofectamine-2000 (Invitrogen). Luciferase activity was detected by the dual luciferase reporter assay system (Promega) following the manufacturer’s manual.

### NF-κB activity assay

In order to clarify that the activity of NF-κB was modulated by miR-23b via targeting A20, THP-1 derived macrophages were transfected or co-transfected with miR-23b inhibitor (or its negative control, NC) or si-A20, or treated with Bay (inhibitor of NF-κB). After cell transfection for 6 h, PMA-differentiated THP-1 cells were incubated with oxLDL for another 24 h, and then the NF-κB activity was examined with an Active NF-κB/p65 ELISA Kit (Imgenex, U.S.A.). Briefly, THP-1 derived macrophages were washed and centrifuged in cold PBS containing PMSF, and then cells were lysed in 750 µl of hypotonic buffer to which 35 µl of detergent solution had been added. After the mixture centrifuged at 14000 ***g*** for 30 s, the supernatant was discarded. About 60 µl of nuclear extraction buffer was added to the pellet for incubation at 4°C for 30 min on a rocking platform, and the supernatant was used as nuclear extract after centrifugation. Microtiter plates coated with p65 antibody were kept at 4°C overnight followed by blocking for 1 h at room temperature with blocking buffer, and then 40 µg of nuclear extract was added to each well and incubated overnight at 4°C. After the plates washed with wash buffer, the amount of bound p65 was quantified by adding alkaline phosphatase-conjugated secondary antibody. The plates were washed and incubated in the presence of para-nitrophenol phosphate as substrate for 30 min at room temperature. Followed by the color development, absorbance values at 405 nm were measured in a microplate ELISA reader (BIO-RAD). NF-κB activity was normalized to the activity of uninfected cells and expressed as fold change.

### Isolation of peripheral blood mononuclear cells

Peripheral blood samples obtained from high cardiovascular risk Mongolian population and healthy Mongolian volunteers were used for peripheral blood mononuclear cells (PBMCs) isolation. PBMCs were isolated by density gradient centrifugation method using Gradisol L (PKutno, Poland). Separation of monocytes was performed with the acceleration of 600 ***g*** for 20 min at RT. Finally, the PBMC layer was collected and washed twice by PBS (250× for 10 min), and then cultured in RPMI 1640 medium in a 5% CO_2_ incubator at 37°C for 2 h. With the non-adherent cells removed, the PBMCs (95% purity) were harvested.

### Statistical analysis

All the statistical data were analyzed by SPSS 20.0 software. The chi-square test (*X*^2^-test) was conducted to analyze the correlation between miR-23b expression and high cardiovascular risk factors. The data were characterized as mean ± SD. Differences between two groups were analyzed using a *T*-test. A value of *P*<0.05 was considered significant.

## Results

### Expression of miR-23b in the Mongolian at high cardiovascular risk

**Figure 1:**
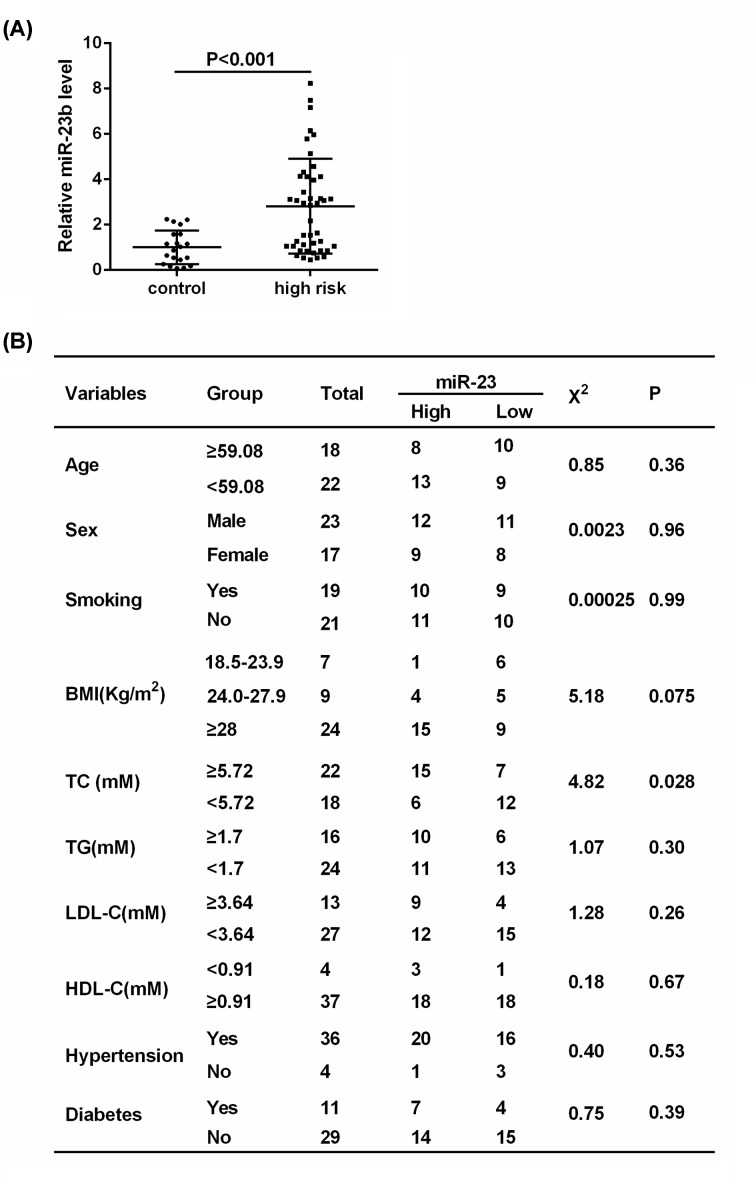
Expression of miR-23b in the Mongolian at high cardiovascular risk and correlation analyses between miR-23b level and cardiovascular risk factors (**A**) The expression of miR-23b in the Mongolian at high cardiovascular risk (*n*=40) and in healthy Mongolian volunteers (control, *n*=20) was determined by qRT-PCR. (**B**) The correlation between miR-23b expression and cardiovascular risk factors was analyzed by chi-square test (*X*^2^-test).

The correlation between miR-23b expression level and high cardiovascular risk was explored. Compared with healthy Mongolian volunteers (control, *n*=20), the expression of miR-23b was significantly higher in the serum of the Mongolian at high cardiovascular risk (high risk, *n*=40) (Figure 1A). The result of *X*^2^-test indicated that miR-23b expression was positively correlated with TC (Figure 1B), which is one of the cardiovascular risk factors. These findings revealed that high expression of miR-23b is closely associated with high cardiovascular risk in the Mongolian.

### Decrease in ABCA1 caused by miR-23b is responsible for TC accumulation

**Figure 2:**
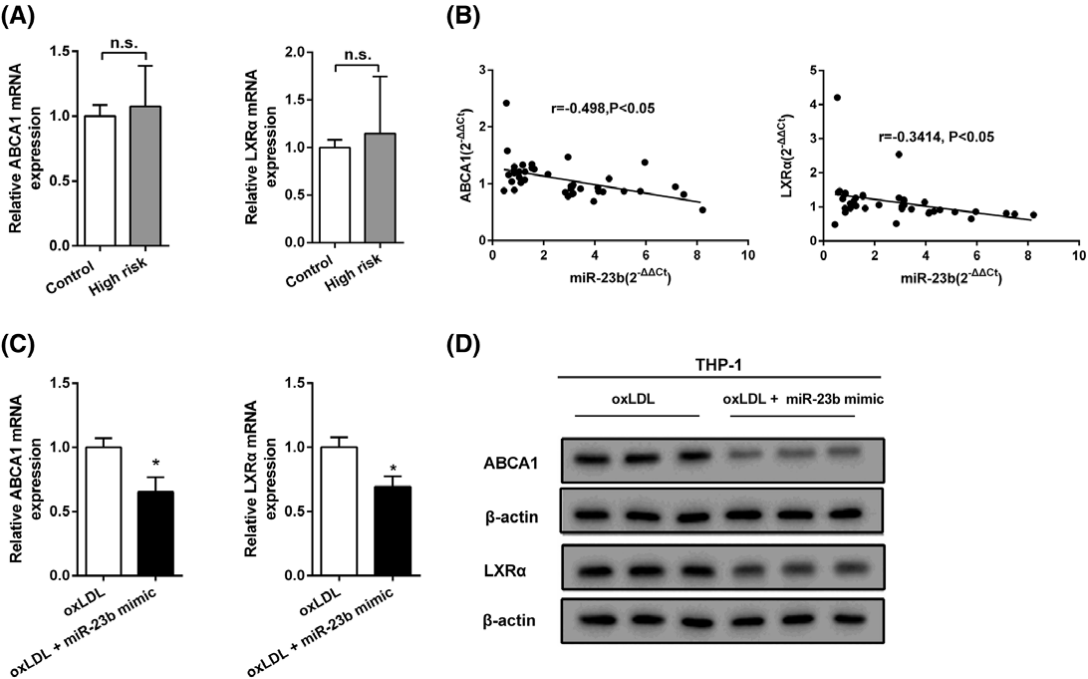
Decrease in ABCA1 caused by miR-23b is responsible for TC accumulation (**A**) Relative expression of ABCA1 and LXRα mRNA in the Mongolian at high cardiovascular risk (*n*=40) and in healthy Mongolian volunteers (control, *n*=20) was quantified with qRT-PCR. (**B**) Spearman correlation analysis was performed to evaluate the association between ABCA1 or LXRα and miR-23b. (**C**) Relative expression of ABCA1 and LXRα mRNA in THP-1 cells was determined by qRT-PCR. (**D**) The protein level of ABCA1 and LXRα in THP-1 cells was assessed by Western blot. *P<0.05 compared with oxLDL.

ATP-binding cassette transporter A1 (ABCA1) is a key modulator of macrophage cholesterol efflux and protects against cholesterol accumulation and atherosclerosis [21], and knockout of Abca1 gene in macrophages promoted pro-inflammatory cytokines expression and activation of NF-κB [22]. Liver X receptor α (LXRα) is a nuclear receptor implicated in cholesterol homeostasis, showing pivotal anti-inflammatory roles in atherosclerotic macrophage [23], and LXRα regulates the reverse transport of cholesterol by up-regulating ABCA1 [24]. Therefore, the expression level of ABCA1 and LXRα in the serum of the Mongolian at high cardiovascular risk was measured to explain the accumulation of TC. As shown in Figure 2A, the expression of ABCA1 and LXRα mRNA was augmented in Mongolian at high cardiovascular risk comparing to the control group, but no significant difference was noted. However, the correlation analyses indicated that miR-23b was negatively correlated with the expression of ABCA1 and LXRα (Figure 2B); moreover, overexpression of miR-23b in oxLDL-treated THP-1 cells markedly restrained the expression levels of ABCA1 and LXRα, both in mRNA (Figure 2C) and protein (Figure 2D). Collectively, high expression of inflammatory factors in oxLDL-treated THP-1 cells largely repressed LXRα expression, which led to reduced ABCA1 expression and inhibited reverse transport of cholesterol, thus contributing to the high level of TC.

### MiR-23b enhances the oxLDL-induced inflammatory response of THP-1 derived macrophage

**Figure 3:**
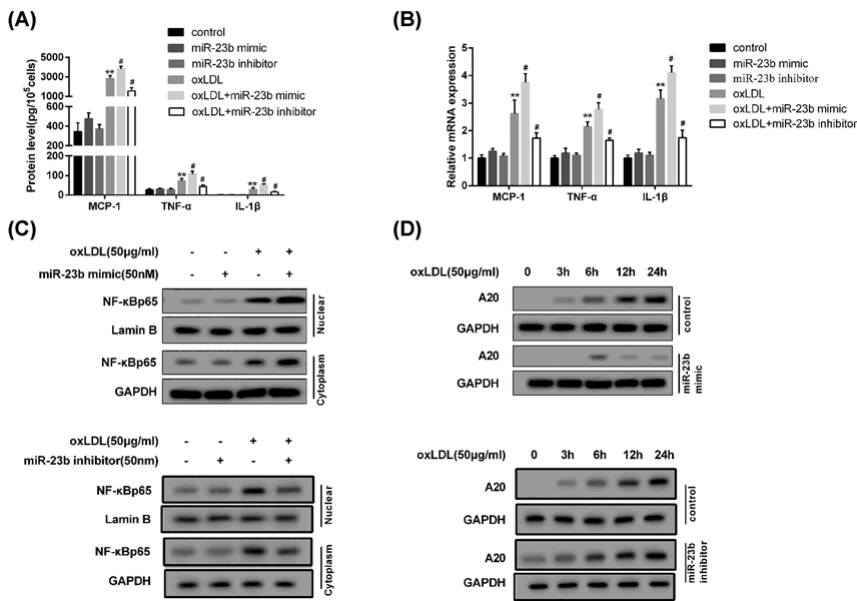
MiR-23b enhanced the oxLDL-induced inflammatory response of THP-1 derived macrophage (**A**) The mRNA expression of MCP-1, TNF-α, and IL-1β in THP-1 derived macrophage inducted by oxLDL was quantified by qRT-PCR. (**B**) The protein level of MCP-1, TNF-α, and IL-1β in THP-1 derived macrophage inducted by oxLDL was measured with ELISA. (**C**) The nuclear and cytoplasmic protein levels of p65 in THP-1 derived macrophage inducted by oxLDL were analyzed by Western blot. (**D**) The protein level of A20 in THP-1 derived macrophage inducted by oxLDL was analyzed by Western blot. **P<0.05 compared with control; #*P*<0.05 compared with oxLDL

To investigate the effect of miR-23b expression on inflammatory reaction of the macrophage differentiated from THP-1 cells, the THP-1 cells were divided into four groups: control, miR-23b mimic, oxLDL, and oxLDL+miR-23b mimic. Compared with the control group, individual use of miR-23b mimic or inhibitor had no notable influence on mRNA and protein level of inflammatory factors (TNF-α and IL-1β) and chemotactic factor (MCP-1) (Figure 3A,B). However, in THP-1 cells treated with oxLDL, miR-23b mimic remarkably promoted the expression of TNF-α, IL-1β, and MCP-1; while miR-23b inhibitor showed the opposite effects (Figure 3A,B). In THP-1 derived macrophages, the cytoplasmic and nuclear p65 levels were all significantly elevated by oxLDL treatment and further boosted by miR-23b mimic; but miR-23b inhibitor reversed the effect of oxLDL on promoting cytoplasmic and nuclear p65 expression (Figure 3C). On the contrary, the expression of A20 protein was clearly reduced by miR-23b mimic but augmented with miR-23b inhibitor transfection (Figure 3D). The above results manifested that miR-23b enhanced the oxLDL-induced inflammatory response and increased the activity of NF-κB in THP-1 derived macrophage.

### MiR-23b activates NF-κB pathway through targeting A20

**Figure 4:**
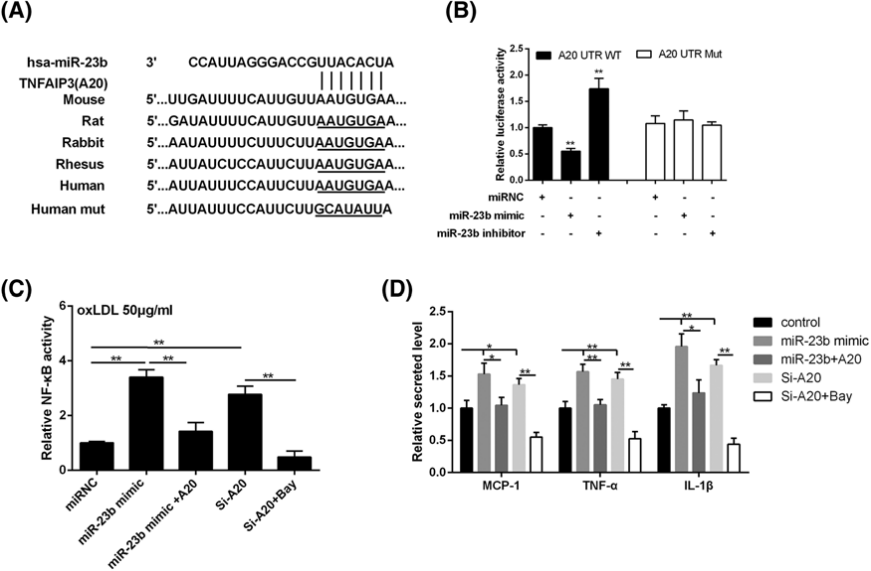
MiR-23b regulated NF-κB pathway through targeting A20 (**A**) The binding site between miR-23b and A20 in different species was predicted by the bioinformatics method (microRNA.org). (**B**) Interaction between miR-23b and A20 was clarified by dual luciferase reporter assay. (**C**) NF-κB p65 translocation into the nucleus was measured by the NF-κB activity detection with an Active NF-κB/p65 ELISA Kit. (**D**) The secretion level of MCP-1, TNF-α, and IL-1β in THP-1 derived macrophage was measured by ELISA. *P<0.05 compared with control or miR-23b mimic; **P<0.05 compared with miRNC.

To investigate the regulatory mechanism of miR-23b on NF-κB, the interaction between miR-23b and A20 was explored. The binding site between miR-23b and A20 was predicted by the bioinformatics method (microRNA.org), and in which the sequence is highly conserved in numerous species (Figure 4A). As compared with that in the miRNC group, the luciferase activity of A20 3’ UTR wild-type (WT) was significantly repressed by miR-23b mimic, but it was clearly inverted by miR-23b inhibitor transfection (Figure 4B). However, the relative luciferase activity in TLR4 3’UTR mutant (Mut) was not affected by miR-23b mimic or inhibitor (Figure 4B). After treated with oxLDL, THP-1 derived macrophages were divided into five groups: negative control (miRNC), miR-23b mimic, miR-23b mimic+si-A20, si-A20, and si-A20+Bay. It revealed that the NF-κB activity was distinctly raised by miR-23b mimic, which was reversed by si-A20 transfection; knockdown of A20 by si-A20 transfection increased NF-κB activity, but it was further inverted with Bay treatment (Figure 4C). The level alteration of TNF-α, IL-1β, and MCP-1 secreted by THP-1 derived macrophages with different treatment was also in accordance with the NF-κB activity (Figure 4D). The above findings proved that miR-23b increased NF-κB activity and NF-κB-induced inflammatory response through targeting A20.

### MiR-23b mediates oxLDL-induced inflammatory response of PBMC in the Mongolian at high cardiovascular risk

**Figure 5:**
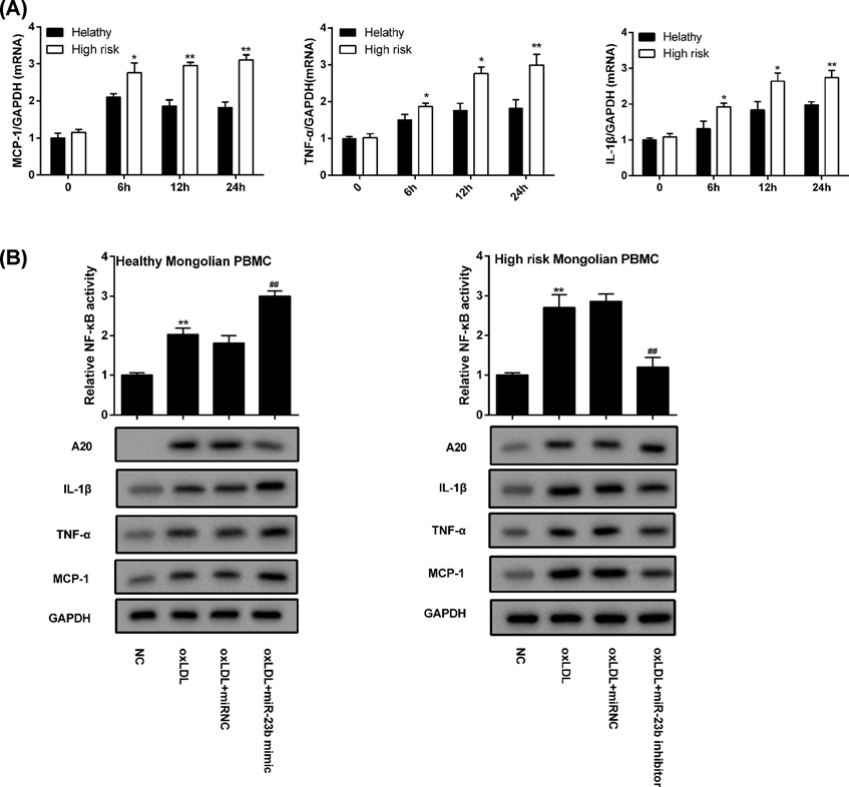
MiR-23b mediated oxLDL-induced inflammatory response of PBMC in the Mongolian at high cardiovascular risk (**A**) The expression of MCP-1, TNF-α, and IL-1β mRNA in PBMC was quantified by qRT-PCR. (**B**) The NF-κB activity in PBMC was detected with an Active NF-κB/p65 ELISA Kit, and the level of A20, IL-1β, TNF-α and MCP-1 protein was analyzed with western blot. *P<0.05 compared with healthy; **P<0.01 compared with healthy; ##*P*<0.05 compared with oxLDL+miRNC

To explore the regulatory effect of miR-23b on PBMCs inflammatory response, PBMCs isolated from the high cardiovascular risk Mongolian population (High risk) and healthy Mongolian volunteers (Healthy) were treated with oxLDL for inflammatory reaction. After treated with oxLDL for 24 h, the expression of TNF-α and MCP-1 at different time was all higher in PBMCs of high risk group (Figure 5A). In PBMCs isolated from healthy Mongolian volunteers (Healthy Mongolian PBMC), the NF-κB activity increased by oxLDL was further elevated by miR-23 mimic, but the expression of A20, TNF-α, IL-1β, and MCP-1 was dramatically decreased (Figure 5B). In PBMCs isolated from the high cardiovascular risk Mongolian population (High risk Mongolian PBMC), the NF-κB activity enhanced by oxLDL was clearly decreased by miR-23 inhibitor, while the expression of A20, TNF-α, IL-1β, and MCP-1 was boosted (Figure 5B). It could be confirmed that miR-23b mediated oxLDL-induced inflammatory response of PBMC via the A20/NF-κB pathway.

## Discussion

In consideration of the high and increasing frequency of CHD and CHD-attributed mortality in the Mongolians, we investigated the expression of miR-23b in serum of the Mongolians at high cardiovascular risk and analyzed the relationship between miR-23b and cardiovascular risk factors. In addition, the role of miR-23b in macrophage inflammation mediated by NF-κB was also focused in the present study. It can be summarized that miR-23b expression was aberrantly high in the Mongolian at high cardiovascular risk, and it was correlated with one of the cardiovascular risk factors, TC; and miR-23b was involved in macrophage inflammatory reaction mediated by NF-κB via targeting A20. The present study clarifies the relationship between miR-23b and high cardiovascular risk in the Mongolians, and highlights its role in macrophage inflammation mediated by NF-κB in atherosclerosis, offering valuable references for atherosclerosis and CHD prevention in Mongolians by avoiding cardiovascular risk factors.

Dysfunction of many types of cells contributes to the development and progression of atherosclerosis, such as migration and proliferation of endothelial and smooth muscle cells, which play critical roles in the maintenance of vascular structure and functions [25]. Endothelial and smooth muscle cells also possess the ability to differentiate into macrophages, which are closely related to inflammation process in atherosclerosis [26]. In the early stage of atherosclerosis, monocyte differentiates into macrophage, and the abnormal intake, synthesis, and efflux of cholesterol in macrophage usually result in lipid deposition and fatty streak formation [27]. Meanwhile, various inflammatory factors secreted by macrophage also exhibit pro-inflammatory effects in atherosclerosis [28]. The present study revealed that the secretion of inflammatory factors (TNF-α and IL-1β) and chemotactic factor (MCP-1) by macrophages was driven by NF-κB, contributing to more severe inflammatory and autoimmune reaction in atherosclerosis progression. Moreover, we demonstrated that the NF-κB signaling was tightly regulated by miR-23b via its well-known inhibitor, A20, shedding light on its molecular mechanism of participating in inflammatory signaling that had been reported previously [29].

Macrophages activation and inflammation have been widely revealed to be regulated by miRNAs, including miR-125a-5p and miR-155 [6,30]. MiR-23b also has been proven to regulate endothelial dysfunction for participating in atherosclerosis progression [31], but its role in macrophage inflammation still remains undetermined. In inflammatory lesions of humans with lupus or rheumatoid arthritis, miR-23b was found to be down-regulated, and it repressed TNF-α-induced NF-κB activation and inflammatory cytokine expression [16]. On the contrary, miR-23b was highly expressed in osteoarthritis-damaged cartilage; and miR-23b level in chondrocyte cells was increased under TNF-α treatment [32]. In these inflammatory diseases, miR-23b exhibited distinct effects on inflammatory reaction, and this may be attributed to the diversity of its target mRNAs. Recently, it has been manifested that suppression of miR-23b prevents cardiovascular dysfunction [33]. Considering the close association between atherosclerosis and cardiovascular disorders, we focused on the expression profile and function of miR-23b in the Mongolian at high cardiovascular risk in the present study. It was illustrated that miR-23b expression was aberrantly high in the Mongolian at high cardiovascular risk; with A20 identified as its target, the regulatory mechanism of miR-23b on macrophage inflammation through NF-κB activation was highly clarified, emphasizing its pro-inflammatory effect in atherosclerosis progression.

Compared with other populations in China, the Mongolian population is mainly located in northern areas of China and has a unique lifestyle and dietary habits, which may be the leading cause of atherosclerosis and CHD [34]. Numerous risk factors of CHD have been verified, including smoking, hypertension, hypercholesterolemia, diabetes, hyperlipidemia, obesity, and drinking, which are depending not only on living habits, but also on ethnic groups [35]. The present study revealed an up-regulation of miR-23b in the Mongolians at high cardiovascular risk and a correlation with the TC, suggesting the relationship among gene expression, racial difference, and lifestyle, and their roles in CHD development. The present study may provide some references for CHD prevention and developing effect therapies in the Mongolian.

In conclusion, the present study demonstrated that abnormally expressed miR-23b in Chinese Mongolian at high cardiovascular risk may promote monocyte/macrophage inflammatory reaction via the A20/NF-κB signaling pathway, and thus contributing to atherosclerosis. With the miR-23 expression Chinese Mongolian at high cardiovascular risk investigated and its impact on inflammatory reaction illuminated, the present study provides novel insight and targets for atherosclerosis and CHD prevention and treatment.

## Abbreviations

ABCA1: ATP-binding cassette transporter A1
CAD: coronary artery disease
CHD: coronary heart disease
IL-1β: interleukin-1β
LXRα: liver X receptor α
MCP-1: monocyte chemoattractant protein-1
NF-κB: nuclear factor-κB
oxLDL: oxidized low-density lipoprotein
PBMC: peripheral blood mononuclear cell
PMI: phorbol 12-myristate 13-acetate
qRT-PCR: quantitative real-time PCR
THP-1: the human monocytic cell line
TNF-α: tumor necrosis factor-α
